# A Review of Embodied Grasping

**DOI:** 10.3390/s25030852

**Published:** 2025-01-30

**Authors:** Jianghao Sun, Pengjun Mao, Lingju Kong, Jun Wang

**Affiliations:** 1 School of Mechanical and Electrical Engineering, Henan University of Science and Technology, Luoyang 471000, China; jianghao@stu.haust.edu.cn (J.S.); kfb@haust.edu.cn (L.K.); 2 School of Information Engineering, Henan University of Science and Technology, Luoyang 471000, China; wj@haust.edu.cn

**Keywords:** pre-trained models, embodied foundation, embodied perception, embodied strategy, embodied agent

## Abstract

Pre-trained models trained with internet-scale data have achieved significant improvements in perception, interaction, and reasoning. Using them as the basis of embodied grasping methods has greatly promoted the development of robotics applications. In this paper, we provide a comprehensive review of the latest developments in this field. First, we summarize the embodied foundations, including cutting-edge embodied robots, simulation platforms, publicly available datasets, and data acquisition methods, to fully understand the research focus. Then, the embodied algorithms are introduced, starting from pre-trained models, with three main research goals: (1) embodied perception, using data captured by visual sensors to perform point cloud extraction or 3D reconstruction, combined with pre-trained models, to understand the target object and external environment and directly predict the execution of actions; (2) embodied strategy: In imitation learning, the pre-trained model is used to enhance data or as a feature extractor to enhance the generalization ability of the model. In reinforcement learning, the pre-trained model is used to obtain the optimal reward function, which improves the learning efficiency and ability of reinforcement learning; (3) embodied agent: The pre-trained model adopts hierarchical or holistic execution to achieve end-to-end robot control. Finally, the challenges of the current research are summarized, and a perspective on feasible technical routes is provided.

## 1. Introduction

In embodied grasping tasks, the embodied robot is the basis for performing the task. Before performing the task, the robot usually needs to be pre-trained on a certain scale of data, so that it can understand human intentions, perceive the surrounding environment fully, and make appropriate decisions when performing the task. The embodied robot then accurately executes the operation instructions and hopefully can learn from the data interacted in real time to improve its adaptability and generalization ability in an unstructured environment.

In recent years, thanks to the development of computing hardware such as GPUs [1] and TPUs [2], deep learning has been applied to large-scale labeled images, videos, texts, and other data in a way that mines data [3] and has achieved a series of breakthroughs in fields such as image recognition [4,5] and language processing [6,7]. Visual foundation models (VFMs) [8] can accurately estimate object categories, poses, and geometric shapes, as well as the spatial relationships between objects, thereby helping the agent make decisions, which allows embodied robots to fully perceive the dynamically complex environment. Large language models (LLMs) [9] enable a robot to better understand language commands from humans and make corresponding decision inferences. Visual–language models (VLMs) [10] combine the advantages of VFMs and LLMs, enabling an agent to reason and make decisions based on task language commands and visual observations of the environment, thereby improving the agent’s perception and understanding of the environment. Generative large models (GLMs) [11] can generate new content such as images and videos based on various data types such as text, images, and videos, thereby enabling richer and more complex creative processes. Robotics domain-specific modes (RDSMs) [12] are trained on specialized datasets, such as human or robotic operation videos, and have better application results.

The breakthroughs in the perception, understanding, and decision-making of pre-trained models have pushed the research of traditional robots in vision-based manipulation [13], reinforcement learning [14], and imitation learning [15] to new heights. In visual-based manipulation and imitation learning, the pre-trained model acts as a visual encoder and text encoder, using its own prior knowledge to improve the efficiency and generalization ability of visual manipulation and imitation learning. The pre-trained model can also generate expert data for imitation learning training in a simulated environment according to specific tasks, alleviating the problem of data scarcity. In reinforcement learning, a large model can generate an appropriate reward function based on an understanding of the task and scenario to guide the generation of reinforcement learning strategies. At the same time, reinforcement learning can be used as a basic strategy for the pre-trained model to continuously optimize the model itself during interaction with the environment. In addition, a new research method has been pioneered, in which a pre-trained model is used as an advanced task planner or to directly output control commands based on sensor information.

**Figure 1:**
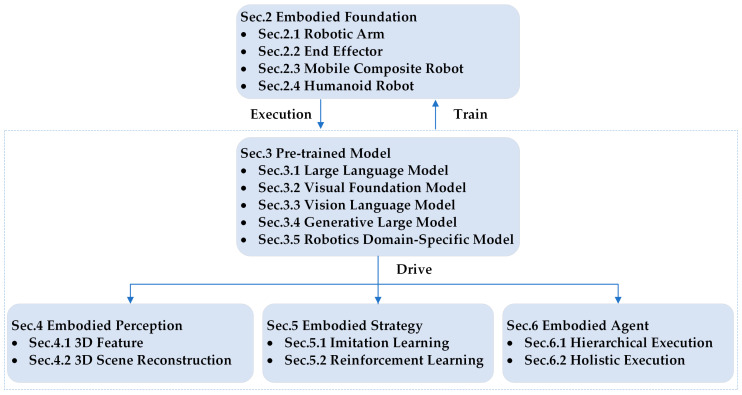
Main organizational framework of this article.

As shown in Figure 1, it is elaborated from five aspects. Among them, embodied perception is the foundation for robots to perceive the external environment. Robots can accurately understand the spatial position information and status of target objects, directly predict actions, or provide input for subsequent decisions. On the basis of perception, embodied strategy generate specific operational strategies through reinforcement learning and imitation learning, guiding robots to make reasonable decisions in complex and dynamic environments. Hierarchical execution within embodied agents integrates perception and strategy through multi-level task planning, ensuring the consistency and robustness of robotic actions. In hierarchical execution, in addition to embodied strategy methods, traditional strategy methods are also incorporated. Overall execution is a fusion of both, achieving global optimization and efficient execution of complex tasks, thereby ensuring the overall consistency and final quality of tasks. Pre-trained models play a crucial driving role in this system. The prior knowledge of large models can help in understanding task objectives, environmental information, and multimodal inputs (such as the combination of vision and language), thereby enhancing the accuracy of perception, the intelligence of strategy generation, and the flexibility of task execution. The required training data come from the embodied foundations, including simulation platforms, open datasets, and data acquisition methods. These data are fully utilized under the impetus of pre-trained models, with the collaboration of three main components: embodied perception, embodied strategy, and embodied agent, ultimately executed by embodied robots in real environments. Section 2 introduces the embodied foundation, including various embodied robots, as well as the corresponding simulation platforms, open datasets, and data acquisition methods. Section 3 introduces five pre-trained models, and Section 4 introduces the embodied perception from the perspectives of 3D features and 3D reconstruction. Section 5 introduces the embodied strategy based on imitation learning and reinforcement learning. Section 6 elaborates on the embodied agent in terms of hierarchical execution and holistic execution. Finally, Section 7 discusses existing challenges and promising future research directions.

The aim of this paper is to provide a review of the research in the field of embodied grasping, with a focus on the application and research progress of traditional two-finger grippers and dexterous hands as end effectors. However, we acknowledge that end effectors also include other types (such as soft grippers, pneumatic grippers, etc.) Although this paper does not delve into these types of end effectors in detail, we believe that the specific structure of end effectors does not fundamentally impact the application of the discussed methods. Therefore, when introducing the end effectors included in the embodied foundation, we not only cover traditional two-finger grippers and dexterous hands but also briefly mention other types of end effector mechanisms.

Comparison with related works: With the rise of large models, various reviews on pre-trained models have emerged [16,17]. However, the integration of pre-trained models and robotics is a relatively new field, and there are few review papers focusing on this area. Existing studies, such as [18,19,20,21], differ significantly from our survey. Yang et al. [18] explored how pre-trained models can be applied to real-world decision-making problems using methods such as prompting, conditional generative modeling, planning, optimal control, and reinforcement learning, while we specifically focus on the application of pre-trained models in robotic grasping and classify the work into three main branches: embodied perception, embodied strategy, and embodied agent. Hu et al. [19] categorized robotic capabilities and investigated systematic ways to enhance these capabilities by integrating pre-trained models, whereas our work focuses on how pre-trained models can be combined with robotic technologies to improve robotic abilities. Xiao et al. [20] reviewed the integration of pre-trained models with traditional robotic learning approaches, covering areas like manipulation, navigation, planning, and reasoning, whereas we concentrate on grasping and provide a more detailed analysis of current techniques in this domain. Finally, Zheng et al. [21] emphasized improving robotic manipulation through physical interaction and sensory feedback, while we highlight the role of pre-trained models in enhancing the learning process itself.

## 2. Embodied Foundation

**Figure 2:**
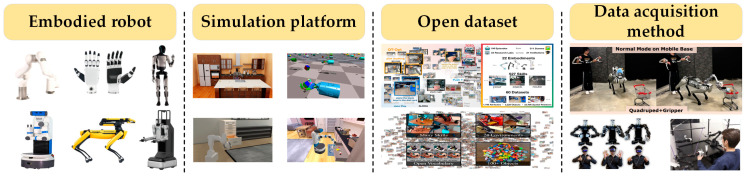
Embodied-foundation content.

As shown in Figure 2, this section will separately introduce commonly used embodied robots, including a single system such as dexterous hand and robotic arm, low-integration mobile complex systems such as wheeled robotic arms and quadrupedal robotic arms, highly integrated mobile complex systems like bipedal humanoid robots and wheeled humanoid robots, as well as corresponding popular simulation platforms, high-quality datasets, and data acquisition methods.

### 2.1. Robotic Arm

Robotic arms were the first robots to be studied, and they have gone through the entire development history of traditional perception and motion control algorithms to intelligent algorithms. They have also experienced the development process from bulky industrial robotic arms to lightweight robotic arms. They are currently widely used in fields such as industrial manufacturing [22] and agricultural harvesting [23]. Commonly used robotic arms include the Franka [24] manufactured by Franka Emika in Munich, Germany, the xArm series [25] produced by UFactory in Shenzhen, China, the UR series [26] developed by Universal Robots in Odense, Denmark, and the ViperX [27] made by Interbotix in the Seattle, WA, USA. In robotic arm research, it is too costly to conduct algorithm research directly on the robot itself. In order to conduct research on artificial intelligence algorithms, researchers have launched a series of simulation platforms, including Gazebo [28], PyBullet [29], and SAPIEN [30], which focus on high-fidelity simulation; RoboSuite [31], ManiSkill series [32,33], and RoboCasa [34], which are optimized for specific tasks or environments; and Isaac Sim [35], which combines commercial applications with advanced GPU computing. Simulation platforms can verify the advantages and disadvantages of the algorithm and make improvements, reducing research costs. Additionally, these platforms also have highly physical characteristics, and the interaction data obtained by robots within them can be used to train embodied-intelligence algorithms. However, due to the existing discrepancies between simulated and real-world environments, models tested on actual robots do not perform as well as the results achieved in simulation environments [36]. With the release of numerous high-quality human-demonstrated robotic arm datasets, research on training robots with real-world datasets has been further developed. Commonly used robotic operation datasets include BridgeData V2 [37], RH20T [38], Open-X [39], and robotic vision datasets RED [40], REGRAD [41], GraspNet-1Billion [42], Grasp-Anything [43]. Additionally, there are datasets focused on specific objects or containing specific types of objects, such as Transpose [44], PokeFlex [45], ClothesNet [46], and SurgT [47]. In terms of constructing custom datasets, UMI [48] utilizes a handheld gripper as the data collection interface, combined with a well-designed interface to collect data on the informative hands and dynamic operation demonstrations in a low-cost and portable manner. ALOHA [49], launched by Stanford University, and GELLO [50], released by the University of California, Berkeley, design active manipulators that can remotely control manipulated arms to directly collect real-world data for imitation learning training. Recently, Stanford University further launched ALOHA2 [51], which not only optimizes the operational performance of the first-generation ALOHA but also integrates remote operation within the Mujoco simulation platform, thereby reducing the cost of data collection. Other methods, including 3D SpaceMouse [52] and RoboTurk [53], can also be employed.

### 2.2. End Effector

Common end effectors include two-finger grippers and dexterous hands [54]. Different end effectors have been designed for various application scenarios, such as pneumatic grippers [55], suction cups [56], jamming [57], Bernoulli [58], and Vortex [59], as well as soft grippers like soft pneumatic grippers [60], cable-driven grippers [61], and hydraulic grippers [62]. Common two-finger grippers include the Robotiq 2F-85 [63] produced by Robotiq in Lévis, Canada and the Franka Emika Gripper [64] made by Franka Emika in Germany. In complex dexterous manipulation scenarios, dexterous hands [65] are a current research hotspot. Due to the fact that they can imitate various functions of human hands, they have great potential in medical services [66,67] and home services [68]. Common dexterous hands include the Allegro Hand [69] made by Allegro Robotics in Seattle, WA, USA, the Shadow Hand [70] manufactured by Shadow Robot in the London, UK, and an open source dexterous hand called Leap Hand [71]. Commonly used simulation platforms for researchers include Isaac Gym [72] and Mujoco [73]. Isaac Gym supports large-scale parallel training for various dexterous manipulation tasks, enabling the rapid accumulation of extensive training data. Additionally, it allows for the exploration of optimal manipulation strategies under different conditions by adjusting simulation parameters. Using hardware such as data gloves [74] and cameras [75] in the real world is also an important method to capture training data. Data gloves can accurately capture the motion and posture of the operator’s hands and turn them into commands to control the end effector. When performing precise operations, data gloves can provide very fine motion control, thereby obtaining high-quality training data. Cameras provide real-time visual information, enabling operators to accurately assess and adjust hand movements to ensure the precise execution of tasks. Common dexterous hand demonstration datasets include UniDexGrasp [76], Handversim [77], and DAPG [78].

### 2.3. Mobile Composite Robot

Mobile composite robots expand the application scenarios of fixed robotic arms. Among them, as a mobile chassis, wheeled robots have a simple structure, are relatively low in cost, and are energy-efficient, allowing for rapid movement on flat surfaces. Quadruped robots can maintain balance and maneuverability on uneven terrain. According to the respective advantages of the two robots, they are combined with robotic arms to form mobile composite robots, which are applied in logistics [79] and disaster relief [80]. Common wheeled composite robots include Fetch Robotics [81] developed by Zebra in Lincolnshire, IL, USA and Hello Robot Stretch [82] from Hello Robot in Martinez, CA, USA. Four-legged composite robots include Spot Arm [83] designed by Boston Dynamics in Waltham, MA, USA, as well as the combination of the quadruped robot B1 and the robotic arm Z1 [84] launched by Unitree in Hangzhou, China. Commonly used simulation platforms include the iGibson series [85,86], the Habitat series [87,88], and AI2-THOR [89], in which the iGibson series also provides data collection and labeling tools, which allow for researchers to conveniently collect behavioral data of robots in a simulated environment and then label and analyze the data. Stanford University has developed the mobile composite robot Mobile ALOHA [90]. In terms of mobility, the robot’s movement speed is close to that of humans, which gives it a significant advantage in scenarios requiring collaboration with humans or tasks executed within human activity spaces. It can also maintain stability when handling large household items and is capable of performing delicate operations. The integrated teleoperation function is a highlight. Through teleoperation, operators can remotely control the robot to carry out tasks and directly collect real-world data in the process.

### 2.4. Humanoid Robot

Compared with other types of robots, humanoid robots are more aligned with human operational behaviors and more adaptable to the diverse scenarios of the human world. As a highly integrated product, they are currently used in research areas such as motor capabilities [91,92] and cognitive operational abilities [93]. In recent years, with the key breakthrough of large models, humanoid robots have ushered in a golden age of rapid development. Representative humanoid robots include Optimus [94] from the American company Tesla, which integrates Tesla’s multiple technological advancements in electric vehicles and artificial intelligence, enabling relatively smooth limb movements. Atlas [95] from the American company Boston Dynamics and H1 [96] from China’s Unitree are renowned for their exceptional mobility performance. Their power systems and balance control capabilities are outstanding. China’s UBTech Walker series [97] and AGIbot Expedition series [98] are unique in terms of intelligence, focusing on deeply integrating artificial intelligence technology into the robot’s behavior decisions. Under traditional control methods, whole-body motion control of humanoid robots presents a complex challenge. Because humanoid robots have multiple degrees of freedom joints, their kinematic and dynamic models are highly intricate. Traditional control algorithms often struggle to precisely coordinate the movements of each joint, leading to insufficient stability. There are limitations in terms of environmental perception. Their limited sensor-data-processing capabilities make it difficult to deeply understand and analyze complex environmental information. Breakthroughs in pre-training models and the application of embodied algorithms have brought new solutions to humanoid robots. Like other robots, simulation training is also the primary method for humanoid robots to learn tasks. Commonly used simulation platforms include Isaac Gym, Mujoco, and BiGym [99]. The AMASS [100] dataset, a motion capture dataset created by the Graphics Laboratory at Carnegie Mellon University, captures a wide range of human movements and actions and is widely used in research in the field of robotics. The University of California, San Diego [101], designed an exoskeleton system that utilizes hand cameras to capture 3D hand gestures and accurately track the position of the end effector through the exoskeleton. In collaboration with the Massachusetts Institute of Technology, they launched a method for collecting data based on VR teleoperation of humanoid robots [102], which offers high real-time performance and stability. Stanford University [103] has developed a low-cost teleoperation method based on visual recognition. It uses visual equipment, such as cameras, to obtain information about the environment and human actions, and the robot operates based on the visual information. This method is low-cost and easy to promote. Embodied robots, simulation platforms, datasets, and data acquisition methods are summarized in Table 1.

## 3. Pre-Trained Model

Pre-trained models have accumulated rich general feature representation capabilities through self-supervised learning on large-scale datasets. They can not only improve the generalization ability of existing models in the robotics field and enable models to better adapt to unknown environments and tasks but also optimize downstream tasks by providing natural language descriptions and prompts.

### 3.1. Large Language Model

In 2018, Google and OpenAI released the BERT [104] with a bidirectional transformer structure and the GPT [105] with a generative pre-trained transformer structure, respectively. In 2019, Google continued to propose a unified text-to-text framework T5 [106]. After several updates and iterations, in 2022, Google once again released PaLM [107], and OpenAI officially launched ChatGPT-3.5 [108]. This model has obtained an accurate understanding of context through pre-training on a large amount of data and has accumulated a wealth of common-sense knowledge about the real world. It also has the ability to solve different tasks through fine-tuning instructions. When it receives a variety of problems, it can flexibly adjust its coping strategies according to the specific needs of the problem based on the knowledge system obtained through pre-training, so as to effectively solve different types of tasks. At the same time, with the help of reinforcement learning optimization training, it can output more positive and positive answers that are in line with human values. Subsequently, in 2023, OpenAI released GPT-4 [9], which increased the model’s context length, which allows it to maintain coherence and accuracy when processing longer text content, while also having the ability to understand multiple modalities. In addition to processing textual information, it can also identify and extract image information, thereby providing users with a more comprehensive and richer interactive experience. GPT-4 has also demonstrated its powerful capabilities, whether it is answering questions about more specialized domain knowledge, or performing complex mathematical reasoning and programming tasks. In 2024, OpenAI released GPT-o1 [109] again, using the Chain-of-Thought (COT) [110] method, which internally generates a detailed chain of thought, just as humans plan and reason first when solving complex problems. First, carefully plan the steps to answer the question, then carry out a rigorous reasoning process, and finally give the final answer. This unique method allows for GPT-o1 to show unprecedented accuracy and efficiency when handling complex tasks.

### 3.2. Visual Foundation Model

In the early stages, the visual field was mainly based on CNN [111] architecture. With the breakthrough of the language model, RNN [112] and transformer [113] in its network architecture were used in the visual field, which has made important progress in image classification [114], object detection [115], and semantic segmentation [116], which further promotes the development of visual pre-training model. Several visual models have been released after GPT. DINO [117] proposes a self-supervised learning method based on knowledge distillation, which ensures the stability and consistency of training. DINOv2 [118] introduces a deeper transformer structure and a more complex attention mechanism based on DINO, significantly improving the model’s expressive power and performance. MAE [119] utilizes visually masked inputs to restore the original image. It can be pre-trained from a large number of unlabeled images, which greatly expands the data resources available for pre-training. SAM [120] is a visual segmentation model. It uses more than 110 million segmentation masks and about 11 million images during training and can accurately segment corresponding regions from images based on given linguistic or visual cues. SAM2 [121] is recently released, trained with more than 51,000 videos and more than 600,000 mask annotations, and improves segmentation accuracy and processing speed based on SAM, as well as supporting object segmentation in videos. Am–radio [122] and Theia [123] are trained by distilling multiple off-the-shelf vision foundation models (VFMs), which results in a smaller model that achieves comparable performance to the larger models while maintaining a smaller model size, reducing the requirements for robot hardware deployment.

### 3.3. Visual–Language Model

LLMs cannot directly understand the output of visual encoders, so they need to convert image encoding into features that the LLM can understand. This is the origin of visual–language models (VLMs), which combine computer vision and natural-language-processing techniques to give models the ability to understand and process image and text data. These models excel at tasks that require simultaneous comprehension of visual content and language and can perform a range of tasks without task-specific training, demonstrating impressive generalization [124]. CLIP [8] uses a large number of paired image and text descriptions as learning materials. The image encoder and text encoder are used to process images and texts, respectively, to extract their respective features, and then these features are optimized in the feature space through contrastive learning [125], enhancing the model’s ability to understand the semantics of images. BLIP [126] and BLIP-2 [10] introduce a curriculum learning strategy that can guide from simpler tasks to more complex tasks, significantly improving performance on tasks such as image captioning and visual question answering. Both Flamingo [127] and GIT [128] pretrain an image encoder through contrastive learning and then perform generative pretraining. PandaGPT [129] and MiniGPT-4 [130] use a single projection layer to achieve visual text alignment, reducing the need to train additional parameters. LLaVa [131], LLaVa2 [132], and KOSMOS-2 [133] are transformer-based causal language models that add the ability to localize and cite. ConvLLaVA [134] uses a hierarchical ConvNeXt as the visual encoder and introduces two key optimization strategies: updating the visual encoder and adding an additional compression stage. Together, these improve the model’s performance on high-resolution inputs.

### 3.4. Generative Large Model

Diffusion [135] models have been used for controllable image generation [136] and conditional image generation of text [137], with the advantages of controllability, conditional generation capabilities, and high-fidelity image generation [138]. DALL-E [139] has learned the complex relationship between text and images through large-scale pretraining and has demonstrated the ability to generate high-quality images from text descriptions. DALL-E 2 [140] proposes a two-stage diffusion model consisting of an a priori algorithm that generates a CLIP image embedding given a text title and a decoder that generates an image based on the encoded image embedding. GLIDE [141] is a text-conditional diffusion model with both CLIP guidance and no-classifier guidance. Make-A-Scene [142] introduces implicit conditions by deriving optional control scene markers from split images and encodes and decodes images and scene markers using two improved Vector-Quantized Variational Autoencoders (VQ-VAEs). IMAGEN [143] is another unsupervised text-conditional diffusion model. Unlike previous methods, it proposes dynamic thresholds to generate more realistic images and a U-Net structure to improve training efficiency. Parti [144] designs a transformer-based autoregressive model that uses ViT-VQGAN as an image encoder to improve the quality of image reconstruction and code utilization. Video-LaVIT [145] predicts the next image or text token through autoregression and processes images and text simultaneously under the unified generation objective. Sora [146] is trained on large-scale video data, including videos and images of varying durations, resolutions, and aspect ratios. It can generate videos not only through text prompts but also by using existing image or video prompts.

### 3.5. Robotics Domain-Specific Model

Pre-trained models, such as CLIP, have been widely used as visual front ends in the robotics field. However, they are trained on large-scale internet data. Although they are very versatile, they usually require dedicated data to obtain exclusive models in specialized fields such as robotics. Such models are more suitable for downstream robotics tasks.

MVP [12] and R3M [147] focus on masked autoencoders and contrastive learning methods, respectively, and perform better in specific types of tasks. VIP [148] is able to generate dense and smooth reward functions for previously unseen robot tasks by combining value function learning and time contrast learning and shows superior performance on multiple tasks, especially in reward function generation and robot control tasks. VC-1 [149] adopts a large version of Vision Transformer (ViT) with more parameters and is trained on a combination of more than 4000 h of human demonstration videos and the ImageNet dataset. Voltron [150] uses videos and corresponding language descriptions based on MVP and R3M to learn representations through linguistic conditional visual reconstruction and visual–language generation. It uses linguistic supervision to improve the recognition of visual patterns and has better generalization capabilities. GR-1 [151] proposes a single GPT-style model that can accept language commands, observe image sequences and robot state sequences as input, and predict robotic actions and future images in an end-to-end manner. After pre-training, it can be fine-tuned on robot data to learn multi-task visual robot manipulation. GR-2 [152] is pre-trained on 38 million video clips, which is one of the largest-scale video pre-trainings used for robotic manipulation agents to date, and a new model architecture has been developed to enable the knowledge gained in the pre-training stage to be seamlessly and losslessly transferred to the fine-tuning stage. SpawnNet [153] has designed an architecture that includes both pre-trained network flows and learnable network flows, enabling the model to combine powerful features of pre-trained and task-specific learning features. In addition to the commonly used datasets, ImageNet [154] and CoCo [155], in the field of vision, there are dedicated datasets in the field of robotics, such as Ego4D [156], Epic-Kitchen [157], and Kinetics-700 [158]. A summary of the pre-trained models is shown in Table 2.

## 4. Embodied Perception

Embodied perception is manifested as an estimation of grasping poses of target objects based on visual sensors. The accuracy of the pose estimation significantly impacts the robot’s ability to successfully grasp the target object, and a robust and efficient pose estimation algorithm needs to be developed. Early grasping tasks were regarded as 2D pose detection [159,160,161], which usually defined the grasping pose as a rectangle from a top-down perspective at a certain height, and predicted the orientation and width of the rectangle. However, due to the lack of 3D geometric information, the predicted grasping pose is limited to 3-DoF. To enable more dexterous grasping by robots, a significant amount of work has focused on 6-DoF grasping, enhancing grasp pose detection [162,163] by directly using depth information, or converting the input RGB-D data into point clouds, then voxelizing the point clouds to generate heatmaps, and estimating the 6-DoF grasp pose under the guidance of the heat maps [164,165,166], or directly inputting them into the pose estimator to generate the pose information required for robotic grasping [167,168]. These methods show high success rates when the depth information is accurate but suffer from degraded performance when encountering photometrically challenging objects (e.g., transparent objects). To alleviate this problem, depth information can be fused with RGB images [169,170].

Other work focus on 3D scene reconstruction to improve the model’s understanding of 3D geometric shapes and then predict the robot’s grasping poses. There are usually two methods: the implicit representation method [171,172], which uses neural rendering and feature distillation to reconstruct 3D feature fields. As for the explicit representation method [173,174], 2D features are reprojected in 3D, and the feature field is optimized. However, these methods all suffer from a lack of semantic information or insufficient generalization.

Pre-trained models possess abundant prior knowledge of visual semantics, and are usually based on point cloud information or 3D scene reconstruction. Combined with traditional 3D visual grasping methods, the use of pre-trained models enhances the ability of visual–language-guided robotic grasping.

### 4.1. Three-Dimensional Feature

**Figure 3:**
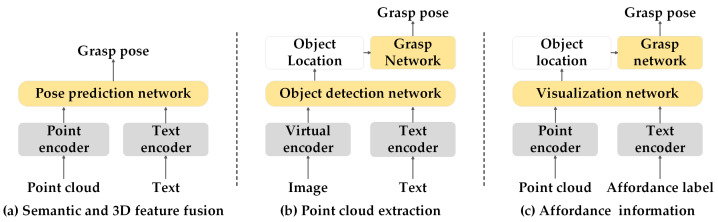
Three-dimensional feature framework.

(1) Semantic and 3D feature fusion: Extracting textual features using large language models and integrating them with point clouds or voxels enables robots to more comprehensively understand and execute robot operation tasks based on natural language instructions, as shown in Figure 3a. Polarnet [175] and Hiveformer [176] apply CLIP to fuse their outputs with point cloud features, while PERACT [177] utilizes CLIP to integrate its outputs with voxel features. Both approaches help the model consider language context when performing action prediction. GraspGPT [178] generates language descriptions of object categories and tasks through a LLM and merges these descriptions with the original dataset to form a new dataset. BERT is used to encode the language descriptions and instructions, combine them with point cloud features to assess the compatibility of the grasping candidates with the task, and predict the final grasping pose. PhyGrasp [179] employs the PointNext framework to convert the point cloud of an object into global and local visual features, and capitalizes Llama 2 to encode the language description of each instance and generate language features. Based on a bridged network, the visual and language features are combined to generate grasping heatmaps and grasping embeddings, which provide the robot with a set of candidate grasping positions.

(2) Point cloud extraction: In the two-stage grasping method, pre-trained models are used to improve visual localization capabilities, which can significantly improve the accuracy of point cloud information extraction, as shown in Figure 3b. VL-Grasp [180] introduces BERT as a text extractor and ResNet as an image extractor in the first stage to predict the 2D bounding boxes and segmentation masks of targets object. The results of the first stage are then used to convert scene-level point clouds into object-level point clouds using a point-cloud-filtering module, followed by the use of a 6-DoF grasp pose detection network to predict the optimal grasp pose. OVGNet [181] leverages GroundingDINO to combine image features and text features for localizing the target object and generating a bounding box. Based on this bounding box, it segments the point cloud belonging to the target object from the complete point cloud data.

(3) Affordance information: Extracting and mapping affordance data from the dataset to achieve zero-shot generalization capabilities for new objects, as shown in Figure 3c. OpenAD [182] jointly learns the visual features of 3D point clouds and the text embeddings of operability labels, leveraging the similarity of text embeddings to achieve zero-shot detection. Robo-abc [183] leverages CLIP to map cropped images of objects into feature vectors, enabling the retrieval of objects from affordance memory that are most visually and semantically similar to a new object. It employs a diffusion model to map the retrieved contact points onto the new object and utilizes AnyGrasp to predict grasp poses based on affordances. Ram [184] extracts unified 2D affordance information from various data sources to construct a comprehensive affordance memory bank. It uses a language model to retrieve tasks that match the given instructions, utilizes feature maps from a visual model to find demonstrations with the most similar viewpoints, then performs 2D affordance transfer, ultimately enhancing 3D affordance.

### 4.2. Three-Dimensional Scene Reconstruction

The focus of 3D scene reconstruction tasks is to enhance the model’s understanding of 3D geometric shapes by converting the input RGB-D data into 3D representations such as NERF or 3DGS.

**Figure 4:**
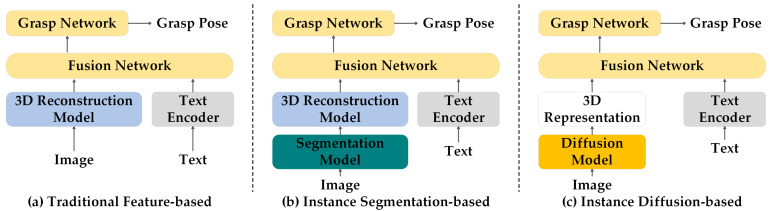
Three-dimensional scene reconstruction framework.

(1) Based on traditional features: Focusing on using open-vocabulary features to understand image content, suitable for scenarios with limited computing resources or where the accuracy of instance segmentation is not critical, as shown in Figure 4a. SPARSEDFF [185] adopts features extracted by DINO to initialize the 3D feature field and designs a lightweight network to solve the problem of local feature differences. F3RM [186] and Splat-MOVER [187] integrate the visual attributes (such as color and lighting effects) and semantic embeddings extracted by CLIP from 2D images with feature fields. LERF-TOGO [188] employs Language Embedded Radiance Fields (LERFs) to combine CLIP’s powerful visual–language capabilities, DINO’s intensive feature extraction capabilities, and 3D scene reconstruction technology to achieve zero-shot task-oriented grasping.

(2) Based on instance segmentation: It provides more refined scene segmentation, suitable for applications that require precise recognition and manipulation of individual objects in the scene as shown in Figure 4b. Object-Aware [189] utilizes GroundedSAM to dynamically segment the camera views and dynamically extract semantic features online to assign semantic labels to each Gaussian point. This endows the scene representation with “object awareness”. GaussianGrasper [190] employs the segmentation priors provided by SAM to accelerate feature field reconstruction and reduce memory usage. CLIP is capable of aligning text descriptions with image content, enabling the mapping of natural language instructions to corresponding visual content. The extracted features are then used to enhance 3D Gaussian primitives.

(3) Based on Diffusion model: Suitable for scenarios that require high resolution and rich semantic information as shown in Figure 4c, GNFactor [191] utilizes Stable Diffusion to extract semantic features from 2D images and designs the Generalizable Neural Feature Fields (GNFs) module to convert these 2D semantic features into representations within 3D space, thereby forming neural feature fields. ManiGaussian [192] introduces a dynamic Gaussian framework and also utilizes Stable Diffusion to extract semantic features from RGB images. Through a Gaussian regressor, the extracted semantic features are mapped into the Gaussian parameter space. This enhances the semantic understanding of the scene representation, enabling robots to comprehend the interactions between objects. A summary of representative algorithms is shown in Table 3.

## 5. Embodied Strategy

Research on embodied strategy primarily focuses on imitation learning and reinforcement learning (RL). Imitation learning achieves skill acquisition by collecting trajectory datasets for specific tasks and using deep neural networks to fit the mapping from time series of states or observations (such as first-person perspective images) to actions [193]. Reinforcement learning, on the other hand, involves the agent learning new skills by interacting directly with the environment and optimizing predefined reward functions related to specific tasks during the interaction [194].

### 5.1. Imitation Learning

Behavior cloning (BC) [195] is the fundamental framework for imitation learning. The loss function of behavioral cloning is expressed as follows:

(1) $$\zeta \left(\theta \right)=-E_{\tau ,l∼D}\left[\sum_{t=0}^{T-1}\log\pi_{\theta }\left(a_{t}|s_{t},l\right)\right]$$

where the robot’s policy representation $\pi \left(a|s,l\right)$ is derived by imitating expert data. The expert dataset is represented as $D={\left\{\tau_{i},l_{i}\right\}}_{i\in [N]}$, in which $\tau_{i}=\left[s_{0},a_{0},s_{1},a_{1},\ldots ,s_{t-1},a_{t-1},s_{T}\right]$ represent the trajectory of experts, and $l_{i}$ represents the task description.

On this basis, imitation learning has been continuously developing in recent years. ACT [151] decomposes complex action sequences into smaller “action blocks” and utilizes Conditional Variational Autoencoders (CVAEs) to learn the latent representations of these blocks. This approach reduces the effective temporal scope of the prediction task, thereby decreasing cumulative errors. During execution, a temporal integration method is employed, generating smooth action sequences by weighted averaging multiple overlapping action predictions, thereby improving the accuracy and stability of the policy. Methods [101,196] have both continued this framework. ATM [197] first utilizes self-supervised learning to pre-train a trajectory model on a large amount of unlabeled videos to predict the future motion trajectories of any point in the video; then, with a small amount of labeled demonstration data, it trains a policy model to learn how to generate control actions based on these predicted trajectories, thereby achieving transfer learning from video demonstrations to actual policies. Diffusion policy [198] first processes the input image sequences through a visual encoder, then uses Denoising Diffusion Probabilistic Models (DDPMs) to simulate the generation process from noise to clear action sequences, and finally optimizes the action sequences through the predicted gradient fields to control the behavior of the robot. Pearce et al. [199] improved the accuracy and reliability of the imitation strategy based on Diffusion-X and Diffusion-KDE as the sampling strategy and designed a network architecture suitable for sequential environments.

The data for imitation learning mainly rely on teleoperation to control the robot to collect data for imitation learning training under specific tasks [200]. However, the high cost of collecting human demonstrations has led to concerns about scaling up the size of the demonstration data. For example, MimicGen [201] has designed a system that takes some expert demonstrations as input and creates an augmented dataset by integrating various scenes and segmenting objects.

Pre-trained models are used in imitation learning in two ways: (1) data augmentation: expand the original dataset, generate expert demonstration data in a simulated environment, or create video sequences to guide the model’s learning; (2) feature extractor, by leveraging the prior knowledge of pre-trained models, improve the model’s ability to adapt to new environments.

#### 5.1.1. Data Augmentation

**Figure 5:**
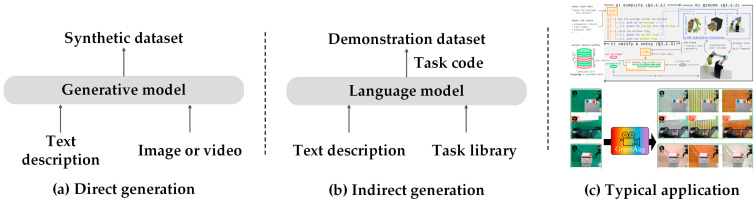
Data augmentation framework and application.

(1) Direct generation: One method is to directly capitalize a generative model to enhance image data, as shown in Figure 5a. GreenAug [202] employs image-text generation models and large-scale image segmentation models to identify objects and backgrounds within images. This approach enables semantic modifications of interactive objects and backgrounds while maintaining the robot’s behavior unchanged, thereby increasing the diversity of the dataset. GenAug [203] provides semantically meaningful data augmentation for imitation learning by leveraging DALL-E 2 and Stable Diffusion in a simulated environment when only a small number of real-world samples are available and expands the dataset by generating diverse visual scenes, including different objects, distractions, and backgrounds. FoAM [204] introduces a fine-tuned vision–language model called InstructPix2Pix (Ip2p) as its Goal Imagination Module. The role of this module is to automatically generate target images, which are then used as conditional inputs to the imitation learning strategy. Another approach is to enrich the dataset of motion trajectories based on diffusion models. xTED [205] adopts diffusion models to edit the data and generate trajectories that are more consistent with the target domain distribution. The edited trajectories are used to train a policy network to generate actions.

(2) Indirect generation: First, tasks are generated using a large language model, and then the dataset is expanded based on these tasks, as shown in Figure 5b. SUaDD [206] capitalizes a large language model for high-level planning and code generation of tasks and generates a large amount of robot data (grasping poses and motion trajectories) with language tags through the combination of a sampling-based planner. GenSim [207] and GenSim2 [208] language models are applied to generate code implementations of tasks that can be executed directly in the simulation environment to generate expert demonstrations. These tasks and demonstrations are used to train robot policies, reducing the reliance on real-world data collection while enhancing the generalization of the policies to new environments and tasks. The typical applications of these two methods are shown in Figure 5c.

#### 5.1.2. Feature Extractor

**Figure 6:**
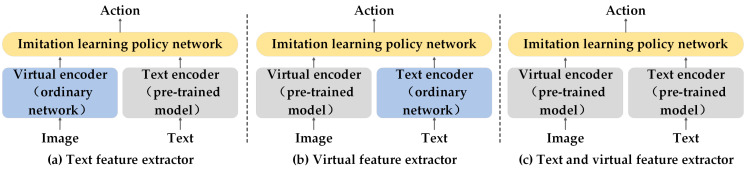
Feature extractor framework.

(1) Text feature extractor, as shown in Figure 6a: MCIL [209] and HULC [210] adopts language models to convert natural language instructions into feature representations in the latent target space, which are then used to train imitation-learning policies. MIDAS [211] enhances the model by adding a residual connection (RC) to the pre-trained language model. It employs a two-stage training process consisting of inverse dynamics pre-training and multi-task fine-tuning. During the inverse dynamics pre-training phase, the model learns to recover action sequences from observed image sequences. In the multi-task fine-tuning phase, the model further learns to execute specific robotic manipulation tasks. RoboCat [212] uses a pre-trained VQ-GAN encoder and CLIP text encoder. RoboCat is trained to mimic the behaviors demonstrated by experts. RoboAgent [213] apply BERT to generate language embeddings for task descriptions. These embeddings are used to condition the transformer policy network, enabling the robot to perform tasks based on natural language instructions. DROID [214] utilizes DistilBERT to convert natural language instructions into feature vectors. The ResNet-50 visual encoder converts each image into a fixed-size feature vector. The diffusion model uses the outputs of the visual and language encoders and the robot’s proprioceptive state to generate action instructions for the robot.

(2) Visual feature extractor, as shown in Figure 6b: EmbCLIP [215] leverages features extracted by CLIP and language instructions as inputs to train a policy network that outputs actions. UMI [48] and DSL [216] extract visual features such as object pose, shape, and location from video data based on the visual foundation model and then apply the extracted features to learn the mapping from observation to action based on a diffusion strategy. HomeRobot [217] can use the visual features extracted by CLIP directly for training strategies or in combination with other modal information (such as depth and semantic segmentation). Vid2robot [218] processes each frame of a video through a visual model to obtain high-dimensional feature representations. The robot’s current state (captured by a camera image) is also encoded using the same image encoder to obtain feature representations similar to those of the prompt video. Actions are then trained using behavior cloning and cross-entropy loss.

(3) Text and visual feature extractor, as shown in Figure 6c: CLIPORT [219] employs a dual-stream architecture, where the semantic stream uses CLIP to process image data and combines it with language instructions, which are fused with the spatial stream and trained using expert demonstrations. VIMA [220] utilizes the T5 to tokenize the input text prompt and then converts these tokens into word embeddings. These embeddings are then fed into the encoder of T5, which outputs a high-dimensional representation of the text that provides the robot agent with contextual information about the task instructions. A Mask R-CNN detector is used to identify objects in the image and extract bounding boxes for the objects and crop the image. The cropped images are divided into fixed-size patches, which are then fed into the ViT model to output visual feature embeddings. These visual embeddings are used together with the bounding-box embeddings to represent the objects in the image. Finally, training is carried out using a behavior-cloning method to imitate the behavior of experts. Open-TeleVision [102] replaces ResNet, used in the ACT algorithm framework, with the more powerful visual backbone network DinoV2 to extract features from the input stereo vision images. SPOC [221] applies DINOv2 and SIGLIP to first extract visual features from images captured by the RGB camera. Then, the CLIP model plays a dual role: on the one hand, it serves as a visual encoder to assist in the extraction of image features, and on the other hand, it is used to process the matching between images and text, helping the model understand and execute text-based instructions. Finally, T5, as a text encoder, specifically processes natural language instructions and converts the text information into a format that the model can execute. These vectors are used to guide the behavior of the agent, enabling it to imitate the expert’s behavior to perform the task. MPI [222] utilizes deformable attention layers to capture the causal relationships between two initial states and the final state and generates aggregated tokens of visual and language embeddings through Multi-Headed Attention Pooling (MAP). SCR [223] extracts feature maps from the middle layer of the Stable Diffusion model and combines them into a final feature representation using a spatial aggregation method. A policy network is trained using supervised learning (such as behavior cloning) to predict the optimal action. RDT [224] provides an algorithmic framework for developing and training foundational robot models for two-handed manipulation tasks. This framework includes mechanisms for handling multimodal inputs and for capturing the multimodality of action distributions through diffusion models.

### 5.2. Reinforcement Learning

By modeling the policy-learning process as a Markov decision process (MDP), the aim is to find a state–behavior mapping function that maximizes the total expected reward of the agent [225]:

(2) $$J\left(\pi \right)=E_{\tau ∼\pi }\left[\sum_{t=0}^{T}\gamma^{t}r_{t}\left(v_{t},a_{t}\right)\right]$$

where $\tau ={\left\{\left(v_{t},a_{t}\right)\right\}}_{t=0}^{T}$ represent the trajectory, and $v_{t}$ and $a_{t}$ denote observation and action at time step t, respectively. The reward r corresponds to the reward provided as environmental feedback after each action is completed, and $\gamma \in \left[0,1\right]$ is a discount factor that balances the importance of the current reward and future rewards. Therefore, the objective of RL can be expressed as follows:

(3) $$\pi^{*}=\underset{\pi }{\arg\max}J\left(\pi \right)$$

Commonly used algorithms for reinforcement learning include DDPG [226], AC series [227,228,229], PPO [230,231], SAC [232], etc. Among them, PPO is widely used in robotic manipulation because of its simplicity and effectiveness. In reinforcement learning, the quality of an agent’s strategy depends on the reward function that is designed. However, the definition of a body-specific reward function often requires some prior knowledge. In current research on embodied intelligence based on reinforcement learning, the reward function is designed manually. Zeng et al. [233] define that the UR5 robot arm successfully pushing the box obtains a reward value of +1, and the reward value is 0 in other cases. Berscheid et al. [234] used the Franka robot arm to perform grasping tasks in cluttered environments, and the reward function was defined as a binary function. Zuo et al. [235] defined the reward function as a function of the distance between the target point and the end-effector of the robot arm. The action of the robot arm is assigned a positive reward as long as it is close to the target point; otherwise, the action is assigned a negative reward. Another approach is to directly learn the reward function itself through human feedback [236], human annotation [237], and behavioral preferences [238].

In the context of large models, because pre-trained large models have a certain prior knowledge of the robot system and state, an optimal reward function can be obtained using a large language model or a visual–language model. This allows the reward function to evolve based on new insights or changes in task requirements, simplifies the solution to complex manipulation tasks, reduces the dependence on manually created reward functions, and potentially improves the effectiveness of the learning process. There are two methods: (1) reward function calculation and (2) reward function estimation.

#### 5.2.1. Reward Function Calculation

**Figure 7:**
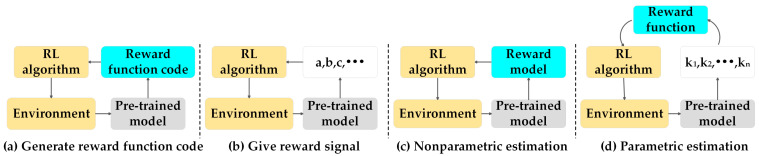
Reward function calculation framework.

(1) Generate reward function code: The generated reward function can be dynamically adjusted according to different task requirements and scenarios, as shown in Figure 7a. Text2Reward [239] represents the elements and states of the environment in the form of Python classes, providing an abstract representation of the environment for the model. It also provides function information and usage examples that help generate reward codes. A large language model is used to generate an intensive reward function that completes the task based on the instruction and the abstract environment. Combined with human feedback, a more refined and effective reward function is iteratively generated. Eureka [240] adopts the GPT-4 to generate an executable reward function using the environmental source code as context. An evolutionary search for rewards is performed within the context window of the LLM by iteratively sampling and improving reward candidate functions. Reward reflection is generated based on the scalar values of each reward component and the task adaptation function at intermediate checkpoints of policy training to guide the improvement of the reward function. ASD [241] employs LLMs to generate task proposals and accordingly defines multiple candidate reward functions. It uses GPT-4V to understand visual scenes and explain the actions of the RL agent, thereby providing an objective assessment of whether the task was successful. The candidate reward functions that are evaluated as successful are retained and further fine-tuned.

(2) Give reward signal: As an evaluation tool, pre-trained visual representations are used to automatically recognize and provide a reward signal, as shown in Figure 7b. ALF [242] applies CLIP to calculate the reward by calculating the similarity score between the observed image after robot execution and the two text prompts (one indicating that the door is closed and the other indicating that the door is open). UVD [243] utilizes VIP, or R3M can help identify the small steps that need to be completed first, generate an embedding vector for each sub-goal, and use the distance between the embedding vector of the current state of the agent and the sub-goal embedding vector as a reward signal. ROBOFUME [244] capitalizes VLMs as reward models and constructs a surrogate reward function that can provide reward signals during the online fine-tuning stage by fine-tuning these models. This reward model can output binary labels for the success state based on the current observation and task name, thereby providing the necessary reward signals when the robot learns a new task. MOKA [245] inputs the description and observation image of the subtask to VLM and prompts it to generate corresponding key points and path points to indirectly estimate the reward signal. RLFP [246] proposes a framework in which GPT-4V serves as the success–reward prior model, used to automatically determine whether a task has been successfully completed and to output corresponding success reward signals (0 or 1). VIP functions as the value base model, accepting current image observations and target image observations as input to infer the value of the state. Seer is an open-source video diffusion model used to generate videos and, through an inverse dynamics model, generate actions to provide inputs for the policy base mode.

#### 5.2.2. Reward Function Estimation

(1) Non-parametric estimation: The form of the reward function is not strictly defined, and a more flexible model is usually used to estimate the reward function, as shown in Figure 7c. MWM [247] designs an auxiliary reward prediction task by adding a linear output head to the autoencoder to predict rewards. VoxPoser [248] leverages a VLM to locate objects of interest in the scene based on the linguistic description of the task. Assign rewards to relevant positions in the observation space; for example, allocate high values to regions of objects that need to be manipulated and low values to regions that should be avoided. Finally, synthesize a 3D value map that includes task-related reward and cost information. These value maps serve as the objective function for the motion planner. LIV [249] estimates rewards by learning a multimodal representation that can implicitly represent the reward function. The distance between the feature representation of the video frame and the feature representation of the target is calculated. The smaller the distance, the closer the content of the video frame is to the target, and the higher the assigned reward value, and vice versa. It can be improved through pretraining and fine-tuning to suit specific tasks and environments. RL-VLM-F [250] queries VLMs to give a preference label for the pair of observed agent-image based on the textual description of the task goal and then learns a reward function consisting of a neural network from these preference labels.

(2) Parameterized estimation: Assuming that the reward function has a specific mathematical form, the reward function is fitted by adjusting predefined parameters, as shown in Figure 7d. CenterGrasp [251] reward function is defined by the weighted sum of a series of terms, each of which is a residual term, and the specific parameters for defining the residual terms are estimated by LLMs. LAMP [252] also adopts an intrinsic incentive function defined by uncertainty in reinforcement learning to guide the agent to efficiently explore the environment. SARU [253] defines the basic structure of the reward function in GPT-4, determines which environmental observation features are relevant to the task and should be included in the reward function, and assigns initial parameter values to each component of the reward function. These parameters are then adjusted during self-alignment to optimize the performance of the reward function. FuRL [254] uses a VLM to generate a reward signal by comparing the cosine similarity between language embeddings (task instructions) and image embeddings (observation of the current state). This reward signal is designed to assist in sparse reward tasks. In terms of video prediction models, VIPER [255] utilizes an autoregressive transformer to train expert videos to obtain a model that can predict the probability distribution of the next frame in a video sequence, with the maximum log likelihood of the next frame given the context as the reward function. Diffusion reward [256] capitalizes expert videos to pretrain a video model. By estimating the conditional entropy of a given historical frame and using its negative value as a reward, the generative diversity of the expert’s behavior can be captured, which better guides the training of the model. A summary of representative algorithms is shown in Table 4.

## 6. Embodied Agent

Model-driven robotic manipulation is a new research approach proposed in recent years. There are two types: (1) Hierarchical execution: High-level task planning is performed for large models, and long-term tasks are decomposed into simpler subtasks. The plan is executed directly using low-level control strategies or a skill library predefined in advance by humans without human intervention; (2) Holistic execution: One is fine-tuning based on a pre-trained model, representing robotic actions as text tags and training with an internet-scale vision–language dataset to directly obtain a VLA (vision–language-action) model, where the robot obtains the task and environmental information to directly output actions. One is visual motion planning, synthesizing video through a pre-trained model and directly controlling the robot with this synthesized video. Actions can also be generated directly using a pre-trained model.

### 6.1. Hierarchical Execution

#### 6.1.1. Low-Level Control Strategy

**Figure 8:**
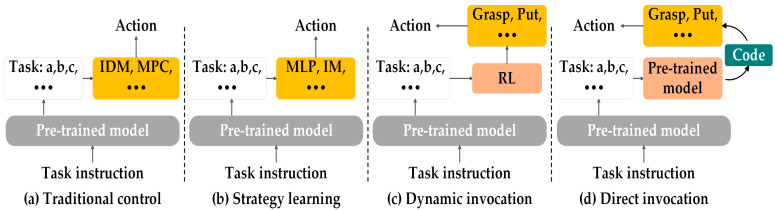
Low-level control strategy and skills library framework.

(1) Traditional control: Stable performance in a known and controlled environment, low computing requirements, suitable for real-time control, as shown in Figure 8a. LLM-GROP [257] applies a LLM to assist the robot in the task of rearranging multiple objects, which is achieved by using the Gazebo simulator. HIP [258] adopts an LLM to generate a symbolic sub-goal sequence, the video diffusion model is used to generate detailed observation trajectories that take into account the geometry and physics of the environment, and the inverse dynamics model is responsible for converting the observation trajectories into specific action instructions. CLOVER [259] utilizes textual conditional video diffusion models to generate visual plans and guide robotic actions. The reliability and accuracy of these plans are enhanced through depth information and optical flow regularization and are executed by a controller designed using inverse dynamics modeling. LMPC [260] leverages PaLM 2 to perform task decomposition, and MPC calculates the specific movement paths and speeds of each joint of the robot based on the actions or strategies provided by the LLM to ensure that the robot can accurately perform these actions. OK-Robot [261] capitalizes OWL-ViT to scan the home environment, generate a 3D map of the environment, and identify the various objects in the environment. After receiving a natural language command, CLIP is first used to convert the command into a semantic embedding, and then the VoxelMap is searched to find the voxel that most closely matches the embedding to determine the location of the target object. After the robot navigates to the vicinity of the target object, Lang-SAM is used to further refine the masked region of the target object so that AnyGrasp can generate a more accurate grasping pose.

(2) Strategy learning: The ability to adapt to complex and dynamically changing environments and handle diverse tasks, as shown in Figure 8b. DEPS [262] introduces a trainable goal selector that selects among parallel candidate subgoals based on how easy they are to achieve and finally generates actions based on the goal condition policy, the current state, and the subgoals. PSL [263] capitalizes GPT-4 to generate high-level plans, and the serialization module is responsible for converting each step in the high-level plan into a target area that the robot needs to reach. Reinforcement learning algorithms are used to learn how to perform specific low-level control actions after reaching the target area. EmbodiedGPT [264] maps feature concrete actions executed by a policy network consisting of a multilayer perceptron (MLP) by combining information from the visual encoder embedding and the planning information provided by the LLM. PALO [265] and YAY Robot [266] adopt pre-trained models to decompose long-term tasks at the semantic level, generating a series of candidate instructions for subtasks. During the training phase, imitation learning is used to learn control strategies from expert demonstrations.

#### 6.1.2. Skills Library

A skills library is a set of predefined actions or action sequences. After a task is broken down, the system can select and combine these actions from the library to complete specific tasks. By combining different actions, diverse task requirements can be met.

(1) Dynamic Invocation: A value function is matched to each skill, which can dynamically adjust the skill selection strategy, as shown in Figure 8c. SayCan [267] utilizes PaLM to break down these high-level instructions into a series of lower-level subtasks or skills. For example, ‘get me a can of Coke’ might be broken down into the subtasks ‘find Coke’, ‘pick up Coke’, and ‘bring it to you’. For each subtask, PaLM evaluates a set of pre-trained skills, which consist of action descriptions and policy networks such as ‘pick up object’ and are trained with imitation learning to obtain an execution policy. A value function is matched to each skill using reinforcement learning, which quantifies the likelihood of successfully executing the skill from the current state and helps select the skill most likely to help complete the current subtask. PaLM-E [268] generates a textual plan based on perceived images and high-level language instructions. In a mobile operating environment, they use SayCan to map the generated plan to executable low-level instructions. When the low-level strategy executes the operation, it can be re-planned according to changes in the environment.

(2) Direct invocation: Generated code invokes the skill library, indirectly invoking the functions defined in the skill library through the generated code, and the designed skill library is suitable for different scenarios and hardware, as shown in Figure 8d. VoicePilot [269] capitalizes GPT-3.5 Turbo for a feeding assistant robot, defining high-level robot control functions that are named in prompts, being able to understand the user’s spoken command generation plan, access these function names, and use them to control the robot. ChatGPT for Robotics [270] and RobotGPT [271] introduce ChatGPT to generate code that instructs the robot to perform a task by calling predefined API functions. These API functions represent primitive actions of the robot. G4R [272] uses GPT-4V to analyze the given RGB video, compile the affordances information, task the plan into a hardware-independent executable file, and save it in JSON format. COME-robot [273] has designed and implemented a complete set of motion primitive libraries, including API functions such as exploration, navigation, and manipulation. It can detect the cause of failure during execution, dynamically request expert feedback, and re-plan actions based on the feedback to resume task execution. LABOR [274] generates a sequence of uncoordinated or coordinated motion commands to coordinate the robot’s hands to complete complex tasks, and adjusts and optimizes this plan through an interactive feedback loop.

### 6.2. Holistic Execution

**Figure 9:**
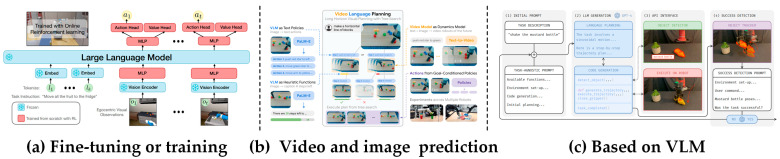
Classic framework for the comprehensive implementation of three methods.

(1) Fine-tuning or training: The entire network is trained or fine-tuned as a whole, with the robot’s movements output directly, and the entire process is completed within a unified framework. RT-1 [275] uniformly discretizes each dimension of the robot’s motion, tokenizes the motion, uses the robot’s state and historical images as input, and directly outputs the motion from the model. LEO [276] uses a two-stage strategy, first performing 3D visual-language alignment and then the 3D visual–language–action command adjustment. RT-2 [277] adopts co-fine-tuning, which fine-tunes both internet-scale visual–linguistic data and robot trajectory data at the same time. This method allows the model to not only learn the robot’s actions but also retain the rich visual and linguistic knowledge it learned during the pre-training stage. LLaRP [278] utilizes LLM as a fixed (frozen) base network, and an adaptation layer is added. The output adaptation layer (action output module) is trained by learning how to adjust its strategy based on sparse rewards provided by the environment through interaction with the environment. OpenVLA [279] employs multiple strategies for fine-tuning and comparison. LLARA [280] fine-tunes the language model and adaptation layer by generating auxiliary command data through self-supervision, without the need for additional action tags. CoGeLoT [281] capitalizes T5 to encode the linguistic part of the command and injects the encoded visual features into the embedding space of T5. The model then outputs an action that defines the linear movement of the robot arm in 3D space, including the start and end postures. DeeR-VLA [282] has proposed an efficient framework capable of dynamically adjusting model size, enabling the real-time execution of complex language-guided tasks on resource-constrained robotic platforms.

(2) Video and image prediction: Action inference is performed by generating a model to generate a video or image. VLP [283] combines PaLM-E and a text-to-video model to generate detailed and rich video and language plans, and uses a goal-conditioned policy to perform the task. DrM [284] leverages a CLIP-Text encoder to encode text descriptions into vector embeddings, which are then used in a conditional video generation model to infer actions by synthesizing videos, which are then used to train a robot policy. Dreamitate [285] applies the Stable Video Diffusion model to receive image input of a new scene and generates a video showing the execution process of the task. The generated video is used for the trajectories of the 3D tracking tool. This trajectory information is converted into movement instructions for the robot, so that the robot can accurately imitate the movements in the video to complete the task. UniPi [286] utilizes the output (text embedding) of T5-XXL as conditional information input to the diffusion model to help generate a video sequence that matches the text description. The action sequence is inferred from the generated video through inverse kinematics modeling. GR-MG [287] generates target images using a target image generation model, and the multimodal target conditional policy uses these target images to predict actions.

(3) Based on VLM: No additional training required, rapid deployment, and efficient processing. ZSTG [288] does not require pre-trained skills, motion primitives, or an external trajectory optimizer. It uses GPT-4 to directly generate high-level plans for robotic manipulation tasks based on task descriptions and predicts a series of dense end-effector poses to guide the robot in completing specific manipulation tasks. KaP [289] designs parser converts the geometric and kinematic structure of an object into a unified text description, including kinematic joint and contact position information. It generates an abstract text operation sequence based on GPT-4, which is further used to generate precise 3D operation points. Chen et al. [290] applies GPT-4o to understand the task and translate it into an action plan that the robot can execute. Then, it generates a series of action instructions based on the task, and uses its spatial reasoning ability to determine how to get from the current state to the target state. KAT [291] utilizes GPT-4 Turbo to convert the visual observations and action sequences in the demonstration into a sequence of tokens and then generates an action sequence. Figure 9 presents the classic framework for implementing the three methods overall. A summary of the algorithms is provided in Table 5.

## 7. Challenges and Prospects

In the past few years, the use of learning-based methods in robotic grasping tasks has increased significantly, promoting rapid development in this field. However, current technology still faces some highly challenging problems. Further exploration of these issues is critical to promoting the widespread use of robots in various fields. This section will discuss several challenges and potential future research directions.

### 7.1. Problems with Dataset Acquisition

Data sources are divided into public datasets and self-built datasets. Data can be obtained from simulation environments or real environments. However, in order to obtain valid data under operational scenarios, it is necessary to remotely control the robot to perform operational tasks based on people. In addition, motion capture of people or animals can also be performed, and the collected data can be used for training by redirection methods. Current simulation platforms still have deficiencies in the simulation of complex flexible objects, fluids, and tactile sensors. There are large gaps in friction, collision, and dynamic interaction and display. At the same time, they face high computational resource consumption, cannot make full use of hardware resources, and lack sufficient environmental diversity. The dataset required for embodied perception is mainly generated in a simulated environment or a combination of simulated and real data. There are differences between the objects in the real environment, which leads to gaps in transfer learning. Moreover, it only covers a limited number of object types and grasping methods, lacking extensive coverage of various objects and complex grasping scenarios, resulting in insufficient generalization. Embodied strategy mainly rely on datasets obtained through robot teleoperation, but teleoperation and manual collection are costly. Open datasets lack a unified format and standards, which makes data integration and comparison more difficult. They may also contain missing data, incorrect labels, or outdated information, which affects the reliability of research results. Future research needs to develop more realistic simulation platforms while optimizing their computational efficiency to reduce resource consumption. Advanced transfer learning methods can be explored to narrow the gap between simulated environments and real-world environments. Large-scale datasets covering diverse object shapes, materials, interaction methods, and complex dynamic scenarios should be developed. To address the lack of standardized formats and protocols for public datasets, efforts can be made to promote the establishment of cross-domain open dataset standards in the future, including data formats and annotation specifications. The direct application of large models to generate datasets, as an emerging approach, has made some progress, but further improvements are still needed in acquiring high-quality datasets and achieving efficient code generation.

### 7.2. Adaptation Problems in Realistic Tasks of Models

Current research is mainly limited to specific tasks and usually achieves good test results in simulation environments. However, in real environments, factors such as the accuracy of sensors and environmental noise limit the ability of models to capture the complexity of the real world. Test results have not reached an ideal level, and there is no immediate and effective handling when operations fail. When decomposing tasks in a large model, it is difficult to ensure that the generated subtask sequence not only conforms to semantic logic but can also be effectively executed in a real environment, which increases the probability of task failure. And in real-time applications, it is necessary to quickly and accurately complete task decomposition and planning, directly call API interfaces, and it is difficult to meet real-time requirements due to transmission rate limitations. Direct deployment requires a model with numerous parameters, which requires a lot of computing resources and makes deployment more difficult. VLA must process and integrate information from multiple modalities, including vision, language, and action. Although significant progress has been made in this area, achieving optimal integration of these modalities remains an ongoing challenge. Future research could introduce diversified simulation scenarios to train models to handle different types of noise, sensor errors, or environmental changes, thereby enhancing their robustness. More efficient large models should be designed, enabling robots to dynamically adjust subtask sequences based on real-time environmental changes to ensure the rationality of task planning. Research into more lightweight model architectures is needed to improve execution efficiency and reduce the computational resource demands of large models, thereby supporting local real-time deployment. For VLA, its multimodal information integration capabilities need to be further enhanced.

### 7.3. Problem of Generalization of Strategies

Existing robotic-grasping technology has insufficient generalization ability when faced with objects of uncommon or special materials or shapes. Robots lack intuitive understanding of physical properties (such as material, density, mass, friction) when grasping, which may lead to improper handling of objects, such as damage when grasping fragile objects. Embodied tasks often involve diverse entity types, and the environment is dynamically changing, such as changes in lighting conditions, movement of surrounding objects, or interference. In this complex situation, after learning the strategy, as long as the dynamic parameters of the agent and the environment change slightly, the original embodied strategy will be difficult to directly apply. This is because existing strategies are often built based on specific training data, which can only cover a limited number of object types, physical properties, and environmental conditions. When faced with new environments or tasks outside the training data, the robot’s existing strategies can hardly achieve effective generalization. For example, a robotic-grasping system trained in a laboratory environment on objects of specific shapes and materials may not be able to accurately grasp objects when applied to actual industrial production scenarios or home environments because the object types and physical properties in the new environment may be quite different from the training data. Future research should focus on addressing the limitations of robotic-grasping generalization. Key areas include enhancing the perception and modeling of physical properties, improving adaptability to dynamic environments, strengthening physical common-sense reasoning, exploring multi-task learning methods, and constructing diversified datasets. Additionally, improving cross-modal data integration and user interaction capabilities will lay a technical foundation for efficient robotic grasping in diverse environments.

### 7.4. Problems in Executing Long Sequence Tasks

Executing long-sequence tasks requires breaking down complex tasks into multiple subtasks and ensuring the logical order of these subtasks. During the execution process, robots need to retain long-term goals and real-time adjust their action plans in response to environmental changes and unexpected events. In this regard, pre-trained models have already achieved initial success, but the continuity of tasks and real-time replanning capabilities are not yet prominent. It is still necessary to integrate various perception and decision-making modules to enhance the overall collaborative capabilities of robots. Future research should continue to focus on the intelligentization of task decomposition, long-term memory and context awareness in task models, real-time response to unexpected events, and deep integration of multimodal perception. Furthermore, enhancing the application of pre-trained models in task planning, with an emphasis on task continuity modeling, failure detection, and recovery mechanisms, will further advance the generalization and adaptability of robots in executing long-sequence tasks.

### 7.5. Interpretability Problem

Despite the rapid development of model applications in robotics, the decision-making process of the model remains a “black box” as the complexity of the model continues to increase, which poses a huge challenge for model debugging and verification. For example, when the robot encounters a sudden obstacle, the model will quickly adjust the trajectory of movement, but it is very challenging to understand how the model makes this adjustment based on previous experience and current environmental changes. Improving the interpretability of the model makes its decision-making process more transparent, which facilitates debugging and verification. Due to the heterogeneity of the data, the model may not be able to effectively extract and integrate all relevant features. This makes it difficult to determine the contribution of each sensor’s data to the final decision when interpreting the model’s decision-making process, which increases the difficulty of interpretability. A better understanding of the dimensions in which existing models do not effectively extract features is needed in order to more effectively collect and utilize data. Future research should focus on improving the interpretability of robotic models, with an emphasis on feature contribution analysis of multimodal data, transparency of decision-making paths, and real-time interpretation of dynamic environments. Additionally, it is necessary to balance the trade-off between model performance and interpretability.

## 8. Conclusions

In this review, we conducted a comprehensive survey of research methods applying foundational models to robotic grasping tasks. We discussed embodied foundations, including robotic systems, simulation platforms, and datasets. Subsequently, we analyzed embodied perception, embodied strategy, and the embodied agent in detail, with a focus on the applications of vision models, language models, vision–language models, and generative models. We summarized the methodologies employed in these studies. Finally, we discussed the remaining challenges in robotics that foundational models have yet to address, as well as promising research directions. We hope this survey will provide researchers with a comprehensive understanding of this emerging field and offer new insights for future exploration.

## Figures and Tables

**Table 1:** Summary of embodied robots, simulation platforms, datasets, and data acquisition methods.

Embodied robots	Robotic arms	Franka [24] xArm series [25] UR series [26] ViperX [27]
	End Effectors	Robotiq 2F-85 [63] Franka Emika Gripper [64] Allegro [69] Shadow [70] Leap [71]
	Mobile composite robots	Fetch Robotics [81] Hello Robot Stretch [82] Spot Arm [83] B1 and Z1 [84]
Humanoid robots		Optimus [94] Atlas [95] H1 [96] Walker series [97] Expedition series [98]
Simulation platforms		Gazebo [28] PyBullet [29] SAPIEN [30] RoboSuite [31] ManiSkill series [32,33] RoboCasa [34] Isaac Sim [35] Isaac Gym [72] Mujoco [73] iGibson series [85,86] Habitat series [87,88] AI2-THOR [89] BiGym [99]
Datasets		BridgeData V2 [37] RH20T [38] Open-X [39] RED [40] REGRAD [41] GraspNet-1Billion [42] Grasp-Anything [43] Transpose [44] PokeFlex [45] ClothesNet [46] SurgT [47] UniDexGrasp [76] Handversim [77] DAPG [78] AMASS [100]
Data acquisition methods		Self-made Equipment [48,49,50,51,90] 3D SpaceMouse [52] RoboTurk [53] Data Gloves [74] Camera [75,103] Exoskeleton System [101] VR [102]

**Table 2:** Representative pre-trained models.

Date	LLM	VFM	VLM	GLM	RDSM
2018	BERT [104] GPT [105]				
2019	T5 [106]				
2020					
2021		DINO [117]	CLIP [8]	DALL-E [139] GLIDE [141]	
2022	PaLM [107] GPT-3.5 [108]		BLIP [126] Flamingo [127] GIT [128]	DALL-E 2 [140] Make-A-Scene [142] IMAGEN [143] Parti [144]	MVP [12] R3M [147] VIP [148]
2023	GPT-4 [9]	DINOv2 [118] SAM [120] Am-radio [122]	BLIP-2 [10] PandaGPT [129] MiniGPT-4 [130] LLaVA [131] LLaVA2 [132] KOSMOS-2 [133] ConvLLaVA [134]	Video LaVIT [145]	VC-1 [149] Voltron [150] GR-1 [151]
2024	GPT-o1 [109]	SAM2 [121] Theia [123]		Sora [146]	GR-2 [152] SpawnNet [153]

**Table 3:** Representative algorithms for embodied perception.

3D feature	Semantic and 3D feature fusion	Polarnet [175] Hiveformer [176] PERACT [177] GraspGPT [178] PhyGrasp [179]
	Point cloud extraction	VL-Grasp [180] OVGNet [181]
	Affordance information	OpenAD [182] Robo-abc [183] Ram [184]
3D scene reconstruction	Based on traditional feature	SPARSEDFF [185] F3RM [186] Splat-MOVER [187] LERF-TOGO [188]
	Based on instance segmentation	Object-Aware [189] GaussianGrasper [190]
	Based on diffusion model	GNFactor [191] ManiGaussian [192]

**Table 4:** Representative algorithms for embodied strategy.

Imitation learning	Data augmentation	Direct generation	GreenAug [202] GenAug [203] FoAM [204] xTED [205]
		Indirect generation	SUaDD [206] GenSim [207] GenSim2 [208]
	Feature extractor	Text feature extractor	MCIL [209] HULC [210] MIDAS [211] RoboCat [212] RoboAgent [213] DROID [214]
		Visual feature extractor	EmbCLIP [215] UMI [48] DSL [216] HomeRobot [217] Vid2robot [218]
		Text and visual feature extractor	CLIPORT [219] VIMA [220] Open-TeleVision [102] SPOC [221] MPI [222] SCR [223] RDT [224]
Reinforcement learning	Reward function calculation	Generate reward function code	Text2Reward [239] Eureka [240] ASD [241]
		Provide reward signal	ALF [242] UVD [243] ROBOFUME [244] MOKA [245] RLFP [246]
	Reward function estimation	Non-parametric estimation	MWM [247] VoxPoser [248] LIV [249] RL-VLM-F [250]
		Parametric estimation	CenterGrasp [251] LAMP [252] SARU [253] FuRL [254] VIPER [255] Diffusion Reward [256]

**Table 5:** Representative algorithms for embodied agent.

Hierarchical execution	Low-level control strategy	Traditional control	LLM-GROP [257] HIP [258] CLOVER [259] LMPC [260] OK-Robot [261]
		Strategy learning	DEPS [262] PSL [263] EmbodiedGPT [264] PALO [265] YAY Robot [266]
	Skills library	Dynamic invocation	SayCan [267] PaLM-E [268]
		Direct invocation	VoicePilot [269] ChatGPT for Robotics [270] RobotGPT [271] G4R [272] COME-robot [273] LABOR[274]
Holistic execution	Fine-tuning or training		RT-1 [275] LEO [276] RT-2 [277] LLaRP [278] OpenVLA [279] LLARA [280] CoGeLoT [281] DeeR-VLA [282]
	Video and image prediction		VLP [283] DrM [284] Dreamitate [285] UniPi [286] GR-MG [287]
	Based on VLM		ZSTG [288] KaP [289] Chen et al. [290] KAT [291]

## Data Availability

Data are contained within the article.
